# Responsiveness of the P4, Patient Specific Functional Scale 2.0, Satisfaction and Recovery Index, and SF-12 in Patients With Musculoskeletal Disorders

**DOI:** 10.7759/cureus.71571

**Published:** 2024-10-15

**Authors:** Hiroshi Takasaki, Yusuke Handa, Hiroki Chiba, Tomoya Kitamura

**Affiliations:** 1 Department of Physical Therapy, Saitama Prefectural University, Koshigaya, JPN; 2 Department of Rehabilitation, Minami Shinjuku Orthopedic Clinic, Tokyo, JPN; 3 Graduate School of Rehabilitation Science, Saitama Prefectural University, Koshigaya, JPN; 4 Department of Rehabilitation, Secomedic Hospital, Funabashi, JPN

**Keywords:** functional disability, minimal clinically important difference, musculoskeletal pain, patient reported outcome measures, responsiveness

## Abstract

Background

Recently, estimation of intervention effects has been recommended using minimum important change (MIC). This study aimed to investigate the responsiveness of the four-item pain intensity measure (P4), Patient Specific Functional Scale 2.0 (PSFS 2.0), Satisfaction and Recovery Index (SRI), and physical (PCS) and mental component summary (MCS) scores of the SF-12v2^®^ Health Survey Acute (QualityMetric Inc. & Fukuhara S, Kyoto) in patients with musculoskeletal disorders and to estimate their MICs.

Methodology

Data of outpatients receiving musculoskeletal physical therapy were collected using a survey in multi-center cohorts. The participants completed the first survey before their first physical therapy session and the second survey after the third to seventh sessions. Responsiveness was assessed by investigating the area under the receiver operating characteristic curve (AUC) and by investigating prior hypotheses with correlations between change scores. In the former method, the AUCs of the measures were calculated for discriminating improved cases (i.e., 11-point Global Rating of Change Scale (GRCS) ≥ 2) and non-improved cases (i.e., GRCS < 2). The MIC scores were estimated with GRCS using the predictive modeling method.

Results

Data from 100 participants were analyzed. The PSFS 2.0 satisfied both acceptable responsiveness criteria, the P4 and SRI satisfied the moderate responsiveness criterion of the construct approach only, and the PCS and MCS satisfied both poor responsiveness criteria. The MICs were 1.64, 2.92, 6.16, 4.49, and 1.67 for the P4, PSFS 2.0, SRI, and PCS and MCS scores, respectively.

Conclusion

The PSFS 2.0 has acceptable responsiveness and can be used to determine treatment effects in clinical practice, while the P4 and SRI can also be used in some cases.

## Introduction

The ideal management of musculoskeletal disease has recently been proposed to require 11 components, one of which includes using appropriate outcomes to track patient change and providing patient-centered care [1]. Patient-reported outcomes (PROMs) are especially important when considering patient-centered care, to assess how patients perceive their situation. PROMs are broadly classified into structured PROMs and non-structured PROMs based on their structure. Structured PROMs have the advantage that the questions are answered by all respondents, thus scores can be compared with others to determine the severity of the disease. However, the questions may be irrelevant to some respondents or may not reflect their issues. On the other hand, non-structured PROMs are more in line with the concept of patient-centered care, as patients themselves set the questions. Although this PROM does not allow scores to be compared to others, it is considered to be more responsive to treatment. In recent years, some semi-structured PROMs that combine features of both structured and non-structured PROMs have been developed [2,3], in which all individuals answer the same questions, but the weight of the score varies among individuals.

Various region-specific PROMs have been developed and used for different purposes. However, in clinical settings where time is limited, obtaining multiple PROMs is burdensome for both therapists and patients. Therefore, the importance of non-disease-specific PROMs has been reaffirmed and new ones have been developed [4]. The Health Survey and SF-12 Health Survey (SF-12) are representatives of non-disease-specific PROMs, but their use is costly and difficult to disseminate to the general public. Among the PROMs that are not disease-specific, the following three free PROMs are considered frequently used: (1) the four-item pain intensity measure (P4), which assesses pain intensity; (2) the Patient Specific Functional Scale (PSFS), which assesses subjective functional impairment; and (3) the Satisfaction and Recovery Index (SRI), which is semi-structured and reflects health-related satisfaction. Recently, a paradigm shift has occurred, whereby estimation of intervention effects is recommended using minimum important change (MIC) rather than statistically significant differences, making the identification of MIC a prerequisite for conducting high-quality clinical trials [5].

The P4 is composed of four 11-point numerical pain rating scales for the severity of pain in the morning, afternoon, evening, and during activity over the past two days, respectively [6]. The P4 was created through a question selection process and has been confirmed to have high internal consistency (α > 0.90) [6] and test-retest reliability (r = 0.78-0.80) [6,7], and a single factor loading has been also assured with a confirmatory factor analysis [6]. The P4 has been reported to have higher test-retest reliability and responsiveness than the widely used single-item numerical rating scale [7]. However, the MIC has not been verified to the best of the author's knowledge.

The PSFS asks patients to list the functional impairments that are most important to them and to report their level of impairment. One concern with the PSFS is that it asks patients how they are doing now compared to how they were before the injury, and if they have a long history of injury, it is difficult for them to recall the situation before the injury. Another concern was that it was sometimes difficult for patients to set up the questions themselves. Therefore, the PSFS 2.0, with modified scale labels and a modified listing of potential items, has recently been developed and found to be easier for respondents to answer than the PSFS [8]. The PSFS 2.0 is expected to replace the PSFS and be widely used in research and clinical practice in the future. However, its MIC has not been verified.

The SRI assigns importance and satisfaction to nine items [3]. These SRI items were selected from a focus group of patients with whiplash-associated disorder and a single factor loading has been identified with exploratory factor analysis [3]. The SRI has preliminary evidence of convergent validity to the SF-12 in patients with various musculoskeletal disorders [9]. The SRI is expected to be widely used in clinical practice and research in the future as an alternative to pain and a regional disease-specific PROM, which have been used to determine recovery after treatment [10]. The aim of this study was to investigate the responsiveness of the P4, PSFS 2.0, SRI, and SF-12 in patients with musculoskeletal disorders and to estimate their MICs.

## Materials and methods

Design

We conducted a multi-center prospective cohort study. Written consent was waived by submitting a complete set of questionnaires as this was a paper-based anonymous survey. The study was approved by the institutional research ethics committee (Saitama Prefectural University, No. 22040).

Participants

Participants were recruited from two medical institutions in Japan (Tokyo and Chiba). Inclusion criteria were (1) >17 years of age with Japanese as the first language, (2) those receiving outpatient physical therapy for musculoskeletal disorders from six physical therapists in charge of data collection, including the authors. Exclusion criteria were (1) those with a diagnosis of neurological disorders or cognitive disorders, and (2) those who did not attend their physical therapy follow-up before the second survey.

To understand the characteristics of the participants, the following demographic and general data were collected at the initial physical therapy session: (1) age, (2) sex, (3) symptom location on a body chart with 22 district areas [11], (4) duration of symptoms, which was defined as the period since the last day when the patient did not feel any symptoms for more than one month.

Data were collected from 100 participants from July 2023 to July 2024. The sample size of 100 was determined based on the COnsensus-based Standards for the selection of health Measurement Instruments (COSMIN) [12].

Physical therapists in charge of data collection

Physical therapy was provided by six physical therapists (mean (SD) of clinical experience = 13.7 (7.0) years) who were credential holders in the McKenzie Method® of Mechanical Diagnosis and Therapy® (MDT; McKenzie Institute International, New Zealand). The intervention was not controlled but followed the principles of the MDT, which is a form of conservative management for musculoskeletal disorders: (1) providing an individualized approach based on a biopsychosocial framework emphasizing patient education including correction of inappropriate patient attitude toward pain and active patient participation in treatment decision-making to promote patient self-management skills, and (2) an algorithm of a management strategy based on MDT classifications and load selection with risk management [13].

Procedures

In the first survey, the participants completed a set of questionnaires before their first physical therapy session. Because the MDT management approach can have rapid effects in the short term [14-17], and to reduce biased data toward improved cases, a second survey was conducted after the third to seventh physical therapy session without referring to the first survey except for the PSFS 2.0 [18]. If participants agreed to participate in the study, they submitted the second questionnaire with the first one together in a survey box.

Outcome measures

The following Japanese-language outcomes were taken from the first and second surveys: (1) the P4 (Appendix 1), (2) the PSFS 2.0 [19], (3) the SRI [9], and (4) the SF-12v2 Acute, Japanese Version 2.0 (QualityMetric Inc. & Fukuhara S, Kyoto). An 11-point global rating of change scale (GRCS) (−5, very much worse; 0, unchanged; 5, completely recovered) [20,21] was also collected in the second survey. The P4 mean value was calculated (0-10). A higher score indicates greater pain intensity.

The PSFS 2.0 score is the average of the maximum of the three items listed by each participant at the time of the initial survey on an 11-point scale (0, no difficulty; 10, impossible to perform the activity). The score ranges from 0 to 10, with lower scores indicating better function. The SRI score is calculated using the following formula from scores of importance and satisfaction on nine items, excluding Item 6.

Weighted score = Importance × Satisfaction

SRI score = Sum of weighted scores/Sum of Importance scores × 10

The SRI scores range from 0 to 100, with higher scores indicating greater health-related satisfaction.

The SF-12 is a one-week recall form including 12 items. Eight psychometric properties (physical functioning, physical role, bodily pain, general health, vitality, social functioning, emotional role, and mental health) were calculated. Then, physical component summary (PCS) and mental component summary (MCS) scores were calculated. Each score ranges from 0 to 100, with higher scores indicating better health status and a score of 50 indicating the Japanese standard. GRCS is a single-item PROM. A score of ≥ 2 was considered an improvement [20].

Data analysis

IBM^®^SPSS^®^ Statistics for Windows, version 28.0 (IBM Corp., Armonk, NY), was used for statistical analyses. The significance level was 5%. A descriptive analysis was used to summarize the characteristics of the participants.

Responsiveness was assessed using two methods proposed by COSMIN developers [22]: (1) a criterion approach by investigating the area under the receiver operating characteristic curve (AUC) and (2) a construct approach by investigating prior hypotheses with correlations between change scores. In the former method, the AUCs of the P4, PSFS 2.0, SRI, and SF-12 PCS and MCS scores were calculated for discriminating improved cases (i.e., GRCS ≥ 2) and non-improved cases (i.e., GRCS < 2). An AUC was interpreted as follows: ≤ 0.7, poor responsiveness; > 0.7, acceptable responsiveness [22]. In the latter method, 12 hypotheses for each of the P4, PSFS 2.0, SRI, and SF-12 PCS and MCS scores, were tested using Pearson’s correlation coefficient for normally distributed data and Spearman’s rank correlation coefficient for non-normally distributed data, respectively, tested by the Shapiro-Wilk test. Interpretations were as follows according to a previous study [23]: poor responsiveness, > 50% of hypotheses were rejected; moderate responsiveness, 25%-50% of hypotheses were rejected; acceptable responsiveness, < 25% of hypotheses were rejected. These hypotheses were developed by the authors and based on a previous study [23].

The MIC scores were estimated with GRCS using the predictive modeling method (MIC_pred_), which is more accurate than the conventional receiver operating characteristic curve method [24]. The MIC_pred_ is calculated followed by formulas using logistic regression, using the dichotomized anchor GRCS response to predict whether a patient belongs to the improved or non-improved group using the change scores in the P4, PSFS 2.0, SRI, and SF-12 PCS and MCS as the predictor.

MIC_pred_ = (log [odds (imp)]-C)/β

where log [odds (imp)] = natural logarithm of proportion improved/(1-proportion improved)]; C = intercept; β = regression coefficient.

Further, to adjust the bias in the proportion of improved and non-improved cases, the MICs were adjusted (MIC_adjusted_) using the following formula [25].

MIC_adjusted _= MIC_pred _- (0.090 + 0.103 × Cor) × SD_change _× log [odds (imp)]

where Cor = point biserial correlation between the P4, PSFS 2.0, SRI, and SF-12 change scores and the GRCS score; SD_change_ = standard deviation of the P4, PSFS 2.0, SRI, and SF-12 change scores.

## Results

**Figure 1:**
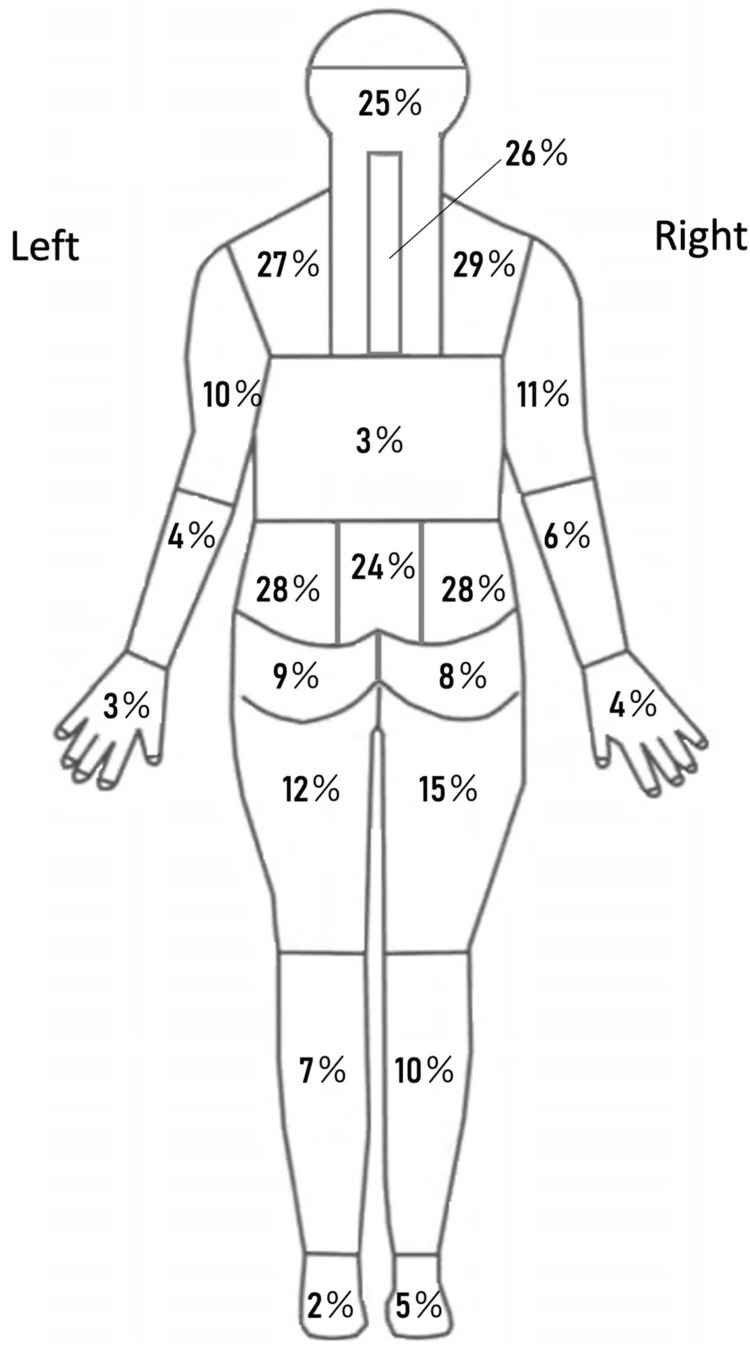
Symptom locations of the participants. Image credits: Hiroshi Takasaki

Characteristics of the 100 participants are summarized in Table 1. Symptom locations are summarized in Figure 1. The mean (SD) interval between the baseline and follow-up sessions was 36.8 (22.9) days. Table 2 shows scores, effect size, and standardized response mean (SRM) that were calculated by dividing the mean change score by the SD of the baseline scores for the P4, PSFS 2.0, SRI, and SF-12 PCS, and MCS scores for the total sample and each GRCS category group. No participant rated a negative GRCS score.

**Table 1:** Baseline characteristics (n = 100). *Values are presented with mean (95% confidence intervals).

Variable	Value
Sex (n of men: n of women)	43: 57
Age (years)*	49.25 (45.66 to 52.84)
Symptom duration, number (≤ 7 days: 8 days to 3 months: ≥ 3 months)	20: 38: 42

**Table 2:** Baseline, follow-up, change scores, effect size, and standardized response mean (SRM) of the P4, PSFS 2.0, SRI, and SF-12 according to the GRCS category. Abbreviations: CIs, confidence intervals; P4, 4-item pain intensity measure; PSFS 2.0, Patient Specific Functional Scale 2.0; SRI, Satisfaction and Recovery Index; PCS, SF-12 physical component summary; MCS, SF-12 mental component summary; GRCS, global rating of change scale. *Improvement of the change score is presented with positive values. The standardized response mean was calculated by dividing the mean change score by the standard deviation of the change.

GRCS category	Number of participants	Measures	Baseline score, mean (95% CIs)	Follow-up mean (95% CIs)	Change score, mean (95% CIs)	Effect size, point estimate (95% CIs)	SRM
Total sample	100	P4	4.13 (3.70 to 4.56)	2.16 (1.74 to 2.58)	1.97 (1.44 to 2.50)*	0.74 (0.52 to 0.96)*	0.74*
		PSFS 2.0	5.85 (5.44 to 6.26)	2.29 (1.88 to 2.70)	3.56 (3.10 to 4.03)	1.53 (1.24 to 1.82)	1.53
		SRI	70.72 (67.80 to 73.64)	77.36 (74.44 to 80.28)	6.64 (4.13 to 9.16)	-0.53 (-0.73 to -0.31)	0.52
		PCS	40.66 (38.51 to 42.81)	45.20 (43.16 to 47.24)	4.54 (2.71 to 6.37)	-0.49 (-0.70 to -0.28)	0.49
		MCS	52.26 (50.51 to 54.02)	55.01 (53.29 to 56.73)	2.75 (1.00 to 4.50)	-0.31 (-0.51 to -0.11)	0.31
+5	12	P4	4.69 (3.10 to 6.28)	1.02 (-0.38 to 2.43)	3.67 (1.75 to 5.58)*	1.22 (0.45 to 1.96)*	1.22*
		PSFS 2.0	5.53 (4.41 to 6.64)	0.50 (-0.21 to 1.21)	5.03 (3.66 to 6.40)	2.34 (1.21 to 3.44)	2.34
		SRI	78.56 (71.09 to 86.02)	88.60 (82.83 to 94.37)	10.04 (3.04 to 17.04)	-0.91 (-1.57 to -0.22)	0.91
		PCS	39.40 (31.47 to 47.33)	45.14 (36.54 to 53.74)	5.74 (-1.57 to 13.06)	-0.50 (-1.09 to 0.11)	0.50
		MCS	56.78 (53.14 to 60.41)	56.56 (50.43 to 62.68)	-0.22 (-4.82 to 4.38)	0.03 (-0.54 to 0.56)	-0.03
+4	36	P4	3.92 (3.16 to 4.67)	1.00 (0.73 to 1.27)	2.92 (2.11 to 3.72)*	1.23 (0.79 to 1.66)*	1.23*
		PSFS 2.0	5.79 (4.98 to 6.60)	1.09 (0.71 to 1.47)	4.70 (3.93 to 5.47)	2.07 (1.48 to 2.65)	2.07
		SRI	73.70 (69.39 to 78.00)	82.49 (78.37 to 86.61)	8.79 (5.16 to 12.43)	-0.82 (-1.19 to -0.44)	0.82
		PCS	43.29 (40.07 to 46.51)	49.46 (47.12 to 51.79)	6.16 (3.16 to 9.17)	-0.69 (-1.06 to -0.33)	0.69
		MCS	51.48 (48.18 to 54.77)	58.25 (55.92 to 60.58)	6.78 (3.99 to 9.56)	-0.82 (-1.20 to -0.44)	0.82
+3	30	P4	3.76 (3.09 to 4.43)	2.54 (1.79 to 3.30)	1.22 (0.46 to 1.97)	0.60 (0.21 to 0.99)	0.60
		PSFS 2.0	5.83 (5.08 to 6.59)	2.87 (2.18 to 3.56)	2.97 (2.35 to 3.58)*	1.81 (1.22 to 2.39)*	1.81*
		SRI	68.03 (62.27 to 73.78)	73.28 (68.25 to 78.30)	5.25 (-0.56 to 11.07)	-0.34 (-0.70 to 0.03)	0.34
		PCS	38.08 (33.86 to 42.30)	43.41 (39.85 to 46.96)	5.33 (1.71 to 8.95)	-0.55 (-0.93 to -0.16)	0.55
		MCS	51.79 (48.65 to 54.92)	53.07 (50.07 to 56.07)	1.28 (-1.82 to 4.38)	-0.15 (-0.51 to 0.21)	0.15
+2	11	P4	4.43 (2.64 to 6.22)	3.14 (2.15 to 4.13)	1.30 (-0.47 to 3.06)*	0.49 (-0.15 to 1.11)*	0.49*
		PSFS 2.0	6.79 (5.62 to 7.95)	4.02 (3.19 to 4.84)	2.77 (1.53 to 4.02)	1.50 (0.60 to 2.36)	1.50
		SRI	66.42 (52.53 to 80.32)	70.15 (58.21 to 82.09)	3.73 (-5.49 to 12.95)	-0.27 (-0.87 to 0.34)	0.27
		PCS	43.72 (38.57 to 48.87)	44.21 (39.18 to 49.24)	0.49 (-4.42 to 5.40)	-0.07 (-0.66 to 0.53)	0.07
		MCS	52.20 (44.96 to 59.44)	53.04 (47.17 to 58.90)	0.84 (-4.89 to 6.57)	-0.10 (-0.69 to 0.50)	0.10
+1	8	P4	4.75 (3.19 to 6.31)	4.53 (2.90 to 6.17)	0.22 (-2.13 to 2.56)*	0.08 (-0.62 to 0.77)*	0.08*
		PSFS 2.0	5.42 (4.05 to 6.79)	4.50 (2.83 to 6.17)	0.92 (0.00 to 1.83)	0.84 (0.00 to 1.63)	0.84
		SRI	66.99 (58.61 to 75.38)	67.94 (56.05 to 79.83)	0.95 (-5.65 to 7.55)	-0.12 (-0.81 to 0.58)	0.12
		PCS	43.65 (36.55 to 50.75)	43.61 (36.54 to 50.68)	-0.04 (-5.40 to 5.32)	0.01 (-0.69 to 0.70)	-0.01
		MCS	50.76 (43.20 to 58.32)	48.18 (37.73 to 58.62)	-2.59 (-11.22 to 6.05)	0.25 (-0.46 to 0.95)	-0.25
0	3	P4	5.50 (-0.49 to 11.49)	6.92 (5.97 to 7.87)	-1.42 (-7.51 to 4.68)*	-0.58 (-1.77 to 0.72)*	-0.58*
		PSFS 2.0	5.78 (-0.33 to 11.88)	5.78 (-0.33 to 11.88)	0.00 (0.00 to 0.00)	0.00 (0.00 to 0.00)	0.00
		SRI	56.16 (32.82 to 79.50)	63.21 (19.22 to 107.20)	7.05 (-29.25 to 43.35)	-0.48 (-1.65 to 0.78)	0.48
		PCS	20.83 (1.15 to 40.51)	20.23 (-17.39 to 57.85)	-0.60 (-18.84 to 7.64)	0.08 (-1.06 to 1.21)	-0.08
		MCS	52.67 (44.62 to 60.71)	54.87 (35.05 to 74.68)	2.20 (-24.64 to 29.04)	-0.20 (-1.33 to 0.97)	0.20

**Figure 2:**
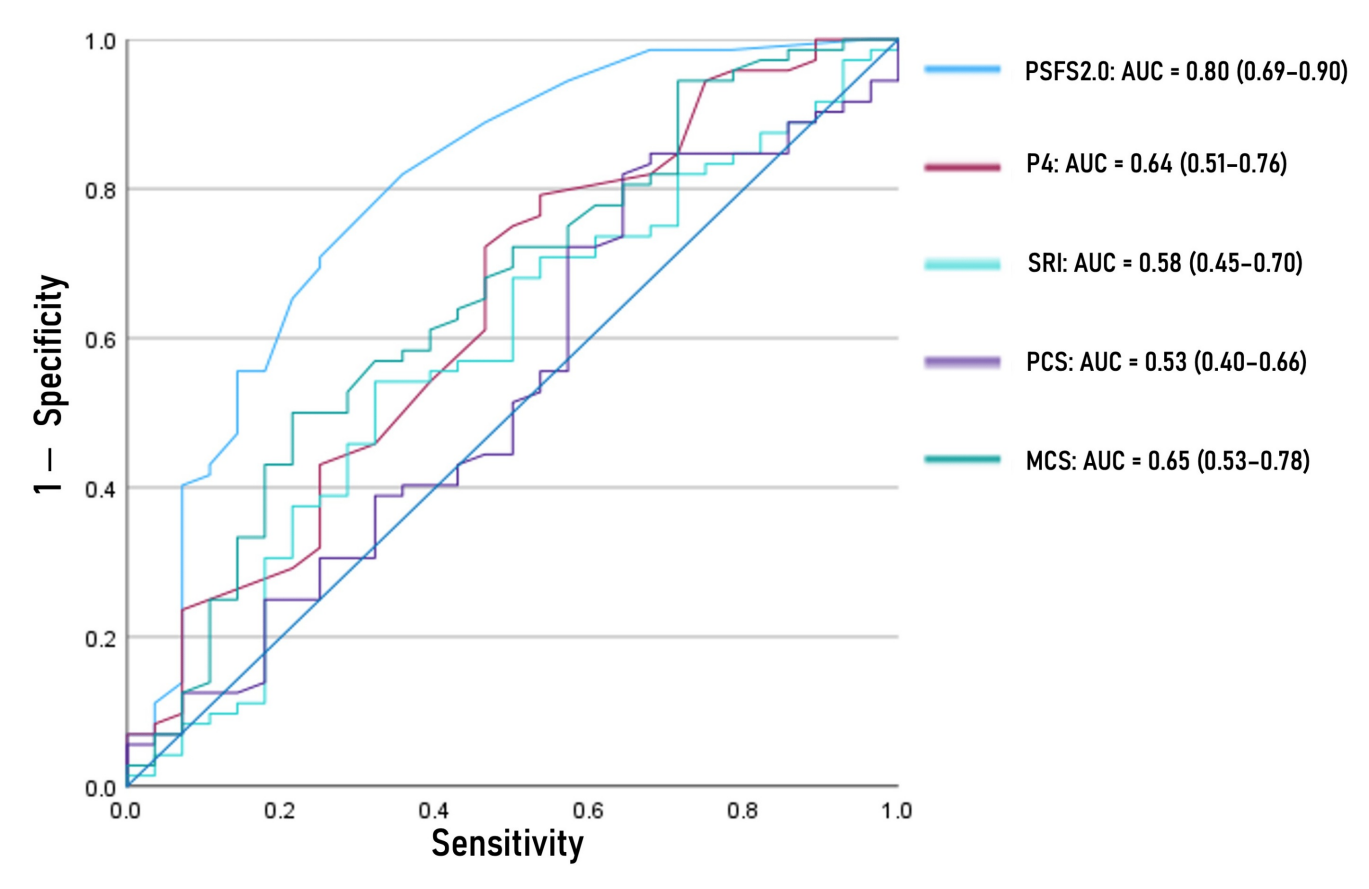
Area under the receiver operating characteristic curves of the five measures. Abbreviation: AUC, Area under the receiver operating characteristic curve; PSFS 2.0, Patient Specific Functional Scale 2.0; P4, 4-item pain intensity measure; SRI, Satisfaction and Recovery Index; PCS, SF-12 physical component summary; MCS, SF-12 mental component summary.

Figure 2 presents AUCs of the five measures, where only PSFS 2.0 has acceptable responsiveness. Table 3 presents results of the construct approach by investigating prior hypotheses with correlations between change scores, where correlations were calculated with the Spearman’s rank correlation coefficient. The PSFS 2.0 was considered to have acceptable responsiveness. The P4 and SRI were considered to have moderate responsive. The SF-12 PCS and MCS were considered to have poor responsiveness. The MIC_adjusted_ values were 1.64, 2.92, 6.16, 4.49, and 1.67 for the P4, PSFS 2.0, SRI, and SF-12 PCS and MCS scores, respectively.

**Table 3:** Results of the construct approach by investigating prior hypotheses with correlations between change scores. Abbreviations: CIs, confidence intervals; NA, not applicable; P4, 4-item pain intensity measure; PSFS 2.0, Patient Specific Functional Scale 2.0; SRI, Satisfaction and Recovery Index; PCS, SF-12 physical component summary; MCS, SF-12 mental component summary; GRCS, global rating of change scale. *Improvement of the change score are presented with positive values. The standardized response mean was calculated by dividing the mean change score by the standard deviation of the change.

Hypotheses	P4	PSFS 2.0	SRI	PCS	MCS
The SRM of the P4*/PSFS 2.0/SRI/PCS/MCS are < 0.2 for patient classifying themselves as “no change” on the GRCS.	✓	✓	✓	✓	✓
The effect size of the P4*/PSFS 2.0/SRI/PCS/MCS are < 0.2 for patient classifying themselves as “no change” on the GRCS.	×	✓	×	×	×
The SRM of the P4*/PSFS 2.0/SRI/PCS/MCS are < 0.2 for patient classified into “non-improvement” on the GRCS.	×	×	×	×	✓
The effect size of the P4*/PSFS 2.0/SRI/PCS/MCS are < 0.2 for patient classified into “non-improvement” on the GRCS.	×	×	×	×	✓
The effect size of the P4*/PSFS 2.0/SRI/PCS/MCS are > 0.5 for patient classifying themselves as “completely recovered” on the GRCS.	✓	✓	✓	×	×
The SRM of the P4*/PSFS 2.0/SRI/PCS/MCS are > 0.5 for patient classifying themselves as “completely recovered” on the GRCS.	✓	✓	✓	×	×
The effect size of the P4*/PSFS 2.0/SRI/PCS/MCS are > 0.5 for patient classifying themselves as “improvement” on the GRCS.	✓	✓	✓	×	×
The SRM of the P4*/PSFS 2.0/SRI/PCS/MCS are > 0.5 for patient classifying themselves as “improvement” on the GRCS.	✓	✓	✓	×	×
The correlation between the P4 change scores and the GRCS is negative and moderate.	✓	NA	NA	NA	NA
The correlation between the P4 change scores and the bodily pain change scores in the SF-12v2 Acute is negative and moderate.	✓	NA	NA	NA	NA
The correlation between the P4 change scores and the bodily pain change scores in the SF-12v2 Acute is lower.	✓	NA	NA	NA	NA
The correlation between the PSFS 2.0 change scores and the GRCS is positive and moderate.	NA	✓	NA	NA	NA
The correlation between the PSFS 2.0 change scores and the physical functioning change scores in the SF-12v2 acute is positive and moderate.	NA	✓	NA	NA	NA
The correlation between the PSFS 2.0 change scores and the PCS change scores is higher (> 0.1) than that between the PSFS 2.0 change scores and the MCS change scores.	NA	✓	NA	NA	NA
The correlation between the PSFS 2.0 change scores and the physical functioning change scores in the SF-12v2 acute is higher (> 0.1) than that between the PSFS 2.0 change scores and the MCS change scores.	NA	✓	NA	NA	NA
The correlation between the SRI change scores and the GRCS is positive and moderate.	NA	NA	×	NA	NA
The correlation between the SRI change scores and the general health change scores in the SF-12v2 acute is positive and moderate.	NA	NA	✓	NA	NA
The correlation between the SRI change scores and the general health change scores in the SF-12v2 acute is higher (> 0.1) than that between the SRI change scores and the PCS change scores.	NA	NA	✓	NA	NA
The correlation between the SRI change scores and the general health change scores in the SF-12v2 acute is higher (> 0.1) than that between the SRI change scores and the MCS change scores.	NA	NA	✓	NA	NA
The correlation between the PCS change scores and the GRCS is positive and moderate.	NA	NA	NA	×	NA
The correlation between the PCS change scores and the physical functioning change scores in the SF-12v2 acute is positive and moderate.	NA	NA	NA	×	NA
The correlation between the PCS change scores and the physical functioning change scores in the SF-12v2 acute is higher (> 0.1) than that between the PCS change scores and the MCS change scores.	NA	NA	NA	✓	NA
The correlation between the PCS change scores and the role physical change scores in the SF-12v2 acute is higher (> 0.1) than that between the PCS change scores and the MCS change scores.	NA	NA	NA	✓	NA
The correlation between the MCS change scores and the GRCS is positive and moderate.	NA	NA	NA	NA	×
The correlation between the MCS change scores and the mental health change scores in the SF-12v2 acute is positive and moderate.	NA	NA	NA	NA	×
The correlation between the MCS change scores and the mental health change scores in the SF-12v2 acute is higher (> 0.1) than that between the MCS change scores and the PCS change scores.	NA	NA	NA	NA	✓
The correlation between the MCS change scores and the vitality change scores in the SF-12v2 acute is higher (> 0.1) than that between the MCS change scores and the PCS change scores.	NA	NA	NA	NA	✓
N (%)	9/12 (75.0)	10/12 (83.3)	8/12 (66.7)	3/12 (25.0)	5/12 (41.7)

## Discussion

In this study, we examined the responsiveness of five outcomes, the P4, PSFS 2.0, SRI, and SF-12, which are representative examples of non-disease-specific outcomes in patients with various musculoskeletal diseases, using two different approaches: a criterion approach and a construct approach. The results showed that the PSFS 2.0 met the criteria for acceptability responsiveness for both methods. Therefore, these results provide evidence for the responsiveness of PSFS 2.0. The P4 and SRI did not meet the criteria in the criterion approach but met the criteria for moderate responsiveness in the construct approach, indicating that there is uncertain evidence that the P4 and SRI display responsiveness. Further, since the SF-12 PCS and MCS did not meet the criteria in the criterion approach and showed poor responsiveness in the construct approach, we can consider that there is sufficient evidence that the SF-12 has poor responsiveness. This finding of higher responsiveness for the PSFS 2.0 and SRI compared to the SF-12 is consistent with the findings of previous studies [3,26]. Therefore, it is recommended that the PSFS 2.0 be used to determine treatment effect in clinical practice, and the P4 and SRI could also be used when pain intensity and recovery after treatment are required to be measured specifically.

In this study, MICs were calculated using a more sophisticated method than previous reports. The MIC value for the PSFS 2.0 was estimated to be 2.92, which is considered to be similar to those reported in patients with upper extremity disorders with a PSFS score of 1.2 [27], those with cervical radiculopathy with a PSFS score of 2.0 [18], those with shoulder pain with a PSFS score of 1.9 to 2.0 [23]. For the SF-12, the PCS values ranged from 1.8 [28] to 8.2 [29], and the MCS values from 1.5 [28] to 10.7 [30] depending on the study. Values for our MICs are within the reported values of these previous studies. On the other hand, no values for MICs have been reported for the P4 and SRI to the author's knowledge. Given the responsiveness results discussed above, the MICs of the PSFS 2.0, P4, and SRI may be widely used in estimating the effects of interventions in future physical therapy clinical trials.

In this study, data were collected at hospitals where intervention by qualified MDTs was available; 42% of the sample had a duration of symptoms for more than three months, but the MCS scores suggest that the study sample did not have major psychological problems. Thus, there could have been a patient selection bias at the stage of choosing a hospital. In the case of chronic pain with major psychological problems, a multidisciplinary team approach is indicated, involving not only physicians and physical therapists, but also clinical psychologists, occupational therapists, and other various professionals. Therefore, the responsiveness and MIC of outcomes examined in this study should be interpreted with caution in those who have major psychological problems and require a multidisciplinary approach.

Limitations

This study has several research limitations. First, while it has the strength that the data of this study can be used to determine the effectiveness of physical therapy because the data were analyzed based on changes before and after physical therapy, caution must be exercised in using the data directly to determine the effectiveness before and after surgery. As a second limitation, since this study was conducted on the Japanese population, caution should be exercised in applying these data directly to different populations.

## Conclusions

In this study, we examined the responsiveness of the P4, PSFS 2.0, SRI, and SF-12 in patients receiving musculoskeletal physical therapy and estimated their MICs. The results showed that PSFS 2.0 has responsiveness, and a difference of three points or more can be used as a criterion for determining clinical effectiveness.

## Appendices

APPENDIX 1: Japanese four-item pain intensity measure (P4)

あなたが治療を受けている問題の痛みについてお答えください。4つの質問に対して、最も適当な痛みの強さ一つにしるしをつけてください。

**Table 4:** Japanese P4 Copyright is obtained from the source [7].

平均すると、痛みの強さはどの程度ですか？	痛みなし										想像出来うる最大の痛み
ここ２日の午前中の痛さ	0	1	2	3	4	5	6	7	8	9	10
ここ２日の午後の痛さ	0	1	2	3	4	5	6	7	8	9	10
ここ２日の夕方の痛さ	0	1	2	3	4	5	6	7	8	9	10
ここ２日のなにか活動時の痛さ	0	1	2	3	4	5	6	7	8	9	10

APPENDIX 2: Original P4

When answering these questions, think only of the pain you are experiencing in relation to the problem for which you are having treatment. Circle 1 number for each of the four questions.

**Table 5:** Original P4 Copyright is obtained from the source [7].

On average, how bad has your pain been	No pain										Pain as bad as it can be
In the morning over the past 2 days?	0	1	2	3	4	5	6	7	8	9	10
In the afternoon over the past 2 days?	0	1	2	3	4	5	6	7	8	9	10
In the evening over the past 2 days?	0	1	2	3	4	5	6	7	8	9	10
With activity over the past 2 days?	0	1	2	3	4	5	6	7	8	9	10
